# Nutritional Characteristics of New Generation Extruded Snack Pellets with Edible Cricket Flour Processed at Various Extrusion Conditions

**DOI:** 10.3390/antiox12061253

**Published:** 2023-06-10

**Authors:** Maciej Combrzyński, Tomasz Oniszczuk, Agnieszka Wójtowicz, Beata Biernacka, Karolina Wojtunik-Kulesza, Maciej Bąkowski, Renata Różyło, Jarosław Szponar, Jakub Soja, Anna Oniszczuk

**Affiliations:** 1 Department of Thermal Technology and Food Process Engineering, University of Life Sciences in Lublin, Głęboka 31, 20-612 Lublin, Poland; tomasz.oniszczuk@up.lublin.pl (T.O.); beata.biernacka@up.lublin.pl (B.B.); jakub.soja@up.lublin.pl (J.S.); 2 Department of Inorganic Chemistry, Medical University of Lublin, Chodźki 4a, 20-093 Lublin, Poland; karolina.wojtunik@umlub.pl (K.W.-K.); anna.oniszczuk@umlub.pl (A.O.); 3 Institute of Animal Nutrition and Bromatology, University of Life Sciences in Lublin, Akademicka 13, 20-950 Lublin, Poland; maciej.bakowski@up.lublin.pl; 4 Department of Food Engineering and Machines, University of Life Sciences in Lublin, Głęboka 28, 20-612 Lublin, Poland; renata.rozylo@up.lublin.pl; 5 Toxicology Clinic, Clinical Department of Toxicology and Cardiology, Medical University of Lublin, Stefan Wyszyński Regional Specialist Hospital, Al. Kraśnicka 100, 20-718 Lublin, Poland; jaroslaw.szponar@umlub.pl

**Keywords:** extrusion-cooking, cricket flour, edible insects, snack pellets, antioxidant activity, polyphenols, physicochemical properties, basic composition, fatty acids

## Abstract

As new sources of proteins, edible insects may be excellent additives in a new generation of environmentally friendly food products that are nutritionally valuable, safe, sustainable, and are needed in today’s world. The aim of this study was to determine the effect of the application of cricket flour on extruded wheat-corn-based snack pellets’ basic composition, fatty acids profile, nutritional value, antioxidant activity and selected physicochemical properties. Results showed that the application of cricket flour had a significant impact on the composition and properties of snack pellets based on wheat-corn blends. In newly developed products, the enhanced level of protein and almost triple increase in crude fiber was found as an insect flour supplementation reached 30% level in the recipe. The level of cricket flour and the applied processing conditions (various moisture contents and screw speeds) significantly affect the water absorption and water solubility index and texture and color profile. Results revealed that cricket flour application significantly increased the total polyphenols content in the assessed samples in comparison to plain wheat-corn bases. Antioxidant activity was also noted to be elevated with increasing cricket flour content. These new types of snack pellets with cricket flour addition may be interesting products with high nutritional value and pro-health properties.

## 1. Introduction

Food and Agriculture Organization of the United Nations promote edible crickets as a way to alleviate the food crisis as more sustainable and environmentally friendly than farming animals, such as chickens, pigs, and cattle [1,2,3]. It is now predicted that the worldwide business focused on edible insects is expected to be 8 billion US $ by 2030 [4]. Insects can be proposed as alternative sources of protein that can be used in various food products [5,6,7,8].

Available data revealed a wide range of nutrient content across edible insect species, with protein levels in some species reaching 81.11 g/100 g [9]. For example, the house cricket, variety *Acheta domesticus* L., is important industrialized insect species in the world [10], and crickets are valuable because they are high in proteins, ranging from 55.0 to 73.0 g/100 g of dry weight, and in lipids ranging from 4.3 to 33.4 g/100 g of dry weight. In particular, cricket powders have been shown to have a protein content of 66.0 g/100 g, a lipid content of 16.0 g/100 g, and an ash content of 5.0 g/100 g, while in farmed crickets, polyunsaturated fatty acids (PUFA) were determined to account for 58% of the total fatty acid content [11,12]. In addition, the in vitro protein digestibility was found to be 76.2%. Zielińska et al. [13] showed the protein content of insect flours as 54.29 g/100 g if mealworm was tested and 71.15 g/100 g if cricket was analyzed. Thus, cricket proteins can be considered comparable in quality to legume proteins (pea and faba bean protein concentrates) [12].

Crickets are also considered rich in macro- and micro-nutrients, such as calcium, magnesium, potassium, sodium, phosphorus, zinc, iron, copper, and manganese, and B group vitamins and A, C, D, E, and K [12]. Proteins derived from crickets have also been noticed as a potential derivation of bioactive peptides useful in the initiation of functional foods, particularly ACE inhibitors [14]. 

Interestingly, the sequestration and metabolization of phenols from the diet have been found to have been mastered by insects in the course of evolution. Selective uptake of quercetin and kaempferol from host plants was observed in the blue butterfly (*Polyommatus icarus*) by Burghardt et al. [15]. Conclusions were drawn based on the study of the content of flavonoids in the mentioned insect. Furthermore, Hirayama et al. [16] identified glycosides of quercetin and kaempferol from the cocoon of the white caterpillar (*Rondotia menciana*) that was fed exclusively with mulberry leaves. These compounds were not identified in the host plant. This indicates that insects can metabolize flavonoids for further incorporation in the cocoon. Ruggeri et al. [17] studied antioxidant activity in a spray-dried (SD) cricket powder, and they noted an increase in radical scavenging activity and gallic acid equivalents, and ferrous ions’ chelating activity and EDTA equivalents up to 24 h. Obtained results revealed releasing of bioactive molecules from cricked powder extracts, which lead to an increased antioxidant activity. Ferreres et al. [18] noted the presence of phenolic acids, such as ferulic, sinapic, and *p*-coumaric, in extracts of larvae of large white butterfly (*Pieris brassicae*) reared on turnips (*Brassica rapa* var. rapa L.). In a follow-up study, ferulic and sinapic acids were also found in this insect when reared on Portuguese cabbage (*Brassica oleracea* var. costata) [19]. Furthermore, the TPC of unprocessed edible beetle (*Eulepida mashona*) resulted in 0.08 g GAE/100 g of the sample [20]. In another study, the total phenolic content of the edible ground cricket (*Henicus whellani*) was found to be 0.77 g GAE/100 g [21], which was lower than the values presented in this study for *A. domesticus*. Cricket flour and its use in functional foods is still in the research phase and is increasingly being proposed as an additive to novel foods [22]. It is worth noting that the European Union prepared a special new legislation on novel foods that settled a procedure for the sale of insect-based foods [23,24]. 

Breeding and transmutation of insects into food ingredients for bundled products are a new denouement. Cricket flour, for instance, has been tested as a raw material in the production of muffins. The test results showed that this flour allows for the baking of a good-quality product [25,26]. Replacing wheat flour with both insect flour and chickpea flour showed a significant positive effect on the rheological properties of dough and bread features. In particular, the replacement of 15% cricket flour increased the stability of the dough and reduced softening degree [22]. 

Little research has been completed on the processing of cricket flour by extrusion. Attempts to use cricket flour to produce meat analogs by extrusion are still underway [27,28]. The goal of researchers and producers is also to estimate the effect of the insertion of cricket powder on the content of proteins, physicochemical characteristics, and extrusion processing conditions of extruded corn crisps. Igual et al. [29] have found that in order to maintain a good quality of directly expanded extrudates, no more than 7.5% of cricket powder can be used. In other studies, snack extrudates of corn flour and cricket powder were fabricated using a single screw extruder at two various temperatures (165 and 175 °C). It was noticed that the introduction of edible insects into extruded snacks could be a good alternative to products available on the market because crickets improve the protein content (with an acceptable share of 5 and 10%) [30]. Moreover, a nutritional analysis of rice-extruded snacks enriched with 10–15% cricket revealed that these formulations are high in protein, fat, dietary fiber, and iron [31]. Extruded potato-based or cereal-based snacks are very popular on the market, but many of these currently have excessive fat, salt, and carbohydrate content, with limited nutritional profits. However, the inclusion of health-promoting components can improve the nutritional potential of this type of product. There are much research about the possibility of using cricket flour in the extrusion processing of snack pellets as half-products to obtain indirectly expanded products ready for further expansion. 

The main subject of this study was to verify the effect of the edible cricket flour addition and variable processing parameters on extruded snack pellets’ basic composition, fatty acid profile, nutritional value, antioxidant activity, and selected physicochemical properties.

## 2. Materials and Methods

### 2.1. Samples Preparation

The basic recipe of the developed pellets was a blend of wheat and corn components (50/50): wheat flour type 450 (supplier: Polskie Młyny Sp. z o.o., Warszawa, Poland) and corn flour (supplier: VIVI Polska Sp. o.o. Sp.k., Grodzisk Mazowiecki, Poland). In the recipes, cricket flour was used as an additive (supplier: SENS Foods, London, UK) at amounts of 10% and 30% of the total blend mass. Blends of dry components were prepared and moistened by mixing with a proper amount of tap water to obtain the 32% and 36% of moisture content. After moistening, the blends were rested for 0.5 h before processing to uniform the moisture distribution. The moisture level was checked before the extrusion-cooking of each blend by applying a standard drying method (1 h at 130 °C). 

**Figure 1:**
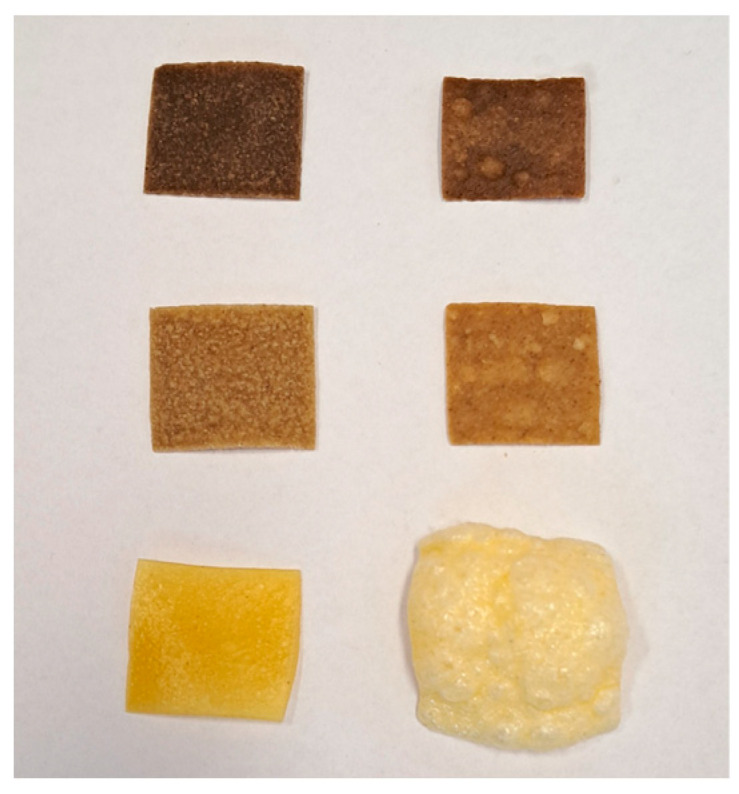
Pellets and expanded snacks with the addition of cricket flour: down—control sample; middle—10% of cricket flour; up—30% of cricket flour.

Processing of snack pellets was completed with a prototype modular single-screw extruder EXP-45-32 (Zamak Mercator, Skawina, Poland) with the configuration L/D = 20 (length of the barrel to screw diameter). Extrusion cooking was performed at 60 and 100 rpm screw rotations. The extrudates (snack pellets) were shaped with a flat die 0.6 × 25 mm and cut by a high-speed roller cutter cooled down by fans to shape the pellets into 25 × 25 mm pieces (Figure 1). Subsequently, the obtained snack pellets were dried in a laboratory shelf dryer at max. 40 °C with forced air circulation to obtain moisture content below 12%. Dried snack pellets stored in dry conditions were ground with an LMN100 (TestChem, Radlin, Poland) laboratory grinder to obtain a 0.3 mm particle size for further testing. 

### 2.2. Pasting Properties

The pasting properties of the raw materials were examined using the Brabender Micro Visco-Amylo-Graph (Brabender, Duisburg, Germany) based on the method reported by Mitrus et al. [32]. Aqueous solutions of 10 g of blends and 100 mL of distilled water were mixed for 5 min before the tests. The measurements were performed at a 250 rpm constant speed and 35 cmg sensitivity. Pasting properties were tested under the following test profile: heating from 30 to 93 °C with the speed of 7.5 °C min^−1^, 5 min holding at 93 °C, cooling from 93 to 50 °C with a speed of 7.5 °C min^−1^, 1 min holding at 50 °C. The 4.1.1 version of Brabender Viscograph software allowed us to determine: peak viscosity (PV)—as maximum viscosity during the heating stage, hot paste viscosity (HPV)—as paste viscosity after 5 min holding at 93 °C, cold paste viscosity (CPV)—as cooked paste viscosity after cooling to 50 °C, breakdown (BD)—the difference between PV and HPV, setback (SB)—the difference between CPV and HPV. Tests were completed in triplicate.

### 2.3. Proximate Composition 

To assess the proximate composition, 250 g of each ground sample was analyzed for dry matter and basic nutrients in accordance with the standards of AACC 1995 [33] and AOAC 2011 [26]. Protein (method 46–10), fat (method 30–10), and ash (method 08–01) contents were appointed in triplicate (AACC 1995) [25]. The fiber content was tested using the 993.21 method (AOAC 2011) [34]. The available carbohydrates were counted as the difference by subtracting the content of dry matter, protein, fat, ash, and fiber from 100.

The composition of fatty acid was measured with the use of the gas chromatography method by utilizing a Varian CP-3800 chromatograph CP-3800 (Varian Inc., Palo Alto, CA, USA) after fats transformation to fatty acids methyl esters (FAME) referring to the 969.33 method of AOAC [35]. The fatty acid separation was completed at the following operating conditions of the chromatograph: 60 m length capillary column CP WAX 52CB DF 0.25 mm, the temperature of column 120 °C increasing gradually by 20 °C/min, 160 °C temperature of feeder, 160 °C temperature of detector, helium as a gas carrier, 127 min time of measurement with 1.4 mL/min of flow rate, and hydrogen and oxygen as other gases. The Supelco 37-Component Fame Mix template (Sigma-Aldrich, Poznań, Poland) was applied. The content of individual fatty acids and their proportion in the total fatty acids value (100%) were received.

### 2.4. Total Phenolics Content (TPC) with Use of Folin–Ciocalteu Method

The ultrasonic bath (Bandelin Electronic GmbH & Co., KG, Berlin, Germany) was used to prepare the extracts of snack pellets using the following conditions: 60 °C temperature and 33 kHz ultrasound frequency at 320 W of power. To get the appropriate extracts, 4 g of ground extrudates were added to 80 mL of methanol (99.8%) and put in an ultrasonic bath for 40 min. After filtration and adding 80 mL of methanol, the extraction process was repeated. After combining both portions of the extracts, they were evaporated to dryness, then dissolved in methanol (10 mL). The following studies were carried out on extracts: free radical scavenging, activity, total phenolics content, and sum of free phenolic acids.

The measurement was performed by applying a modified Folin–Ciocalteu (FC) method [36]. Herein, extract (200 μL) was first combined with distilled water (1.8 mL); subsequently, 200 μL of FC reagent was added, and the combination was then mixed and left for 5 min. After this, 2 mL of 7% Na_2_CO_3_ was added, and the mixture was incubated for 60 min at 40 °C. The Genesys 20 UV-VIST (Thermo Scientific, Waltham, MA, USA) UV-VIS spectrophotometer was applied to measure absorbance at 760 nm. The TPC was presented as μg gallic acid equivalents (GAE) per g of dry mass (d.m.).

### 2.5. DPPH Method Free Radical Scavenging Activity 

The DPPH (2,2-diphenyl-1-picrylhydrazyl) method was used in order to determine the free radical scavenging activity of the obtained extracts. The appropriate volume of extract was added to the DPPH solution (in methanol). The procedure was based on a modified method reported by Burda and Oleszek [37] with the use of a Genesys 20 UV-VIST (Thermo Scientific, Waltham, MA, USA) UV-VIS spectrophotometer working with 517 nm wavelength. The absorbance was measured every 5 min for 30 min immediately after reaction initiation and pure methanol was used for calibration. Tests were performed three times, and results were presented as DPPH scavenging activity and TEAC (Trolox equivalent antioxidant activity) values.

### 2.6. Water Absorption Index (WAI) 

WAI was determined as described by Wójtowicz and Mościcki [38]. The ground pellets (0.7 g) were continuously mixed for 20 min with distilled water (7 mL), and the suspension was then centrifuged for 10 min using a Digicen 21 centrifuge (Labsystem, Kraków, Poland) at 15,000 rpm. Liquid from the obtained gel was collected afterward, and the remained gel was weighed. The water absorption index (WAI) measured in triplicate was counted following the formula:

(1) $$WAI=\frac{m_{g}}{m_{s}}\;[g/g]$$

where m_s_—dry sample mass [g] and m_g_—gel mass [g].

### 2.7. Water Solubility Index (WSI)

Water Solubility Index was determined as described by Wójtowicz and Mościcki [38]. The liquid gained after WAI measurement was evaporated at 110 °C until all water was driven off entirely. WSI measured in triplicate was then calculated using the formula:

(2) $$WSI=\frac{m_{v}-m_{dv}}{m_{s}}\;100\;[\%]$$

where m_dv_—vessel mass after drying [g], m_v_—vessel mass before drying [g], and m_s_—dry sample mass [g].

### 2.8. Cutting Force Measurements

The cutting force of snack pellets was tested with Zwick/Roell apparatus BDO-FB0.5TH (Zwick GmbH & Co., Ulm, Germany) based on Jin et al. [39] methodology. The Warner-Bratzler knife was used for texture assessment, and flat pellets placed on the working table were subjected for cutting with a constant test speed of 500 mm min^−1^. The cutting force was registered as the highest force (N) when the pellet became completely broken. The tests were performed in five replications.

### 2.9. Color Profile of Snack Pellets

The profile of color coordinates in the CIE-Lab scale was evaluated and the total color change index (*ΔE*) using the NR20XE colorimeter (Shenzhen, China). Ground samples of snack pellets were tested for lightness L* ranging from 0 (black) to +100 (white), redness a*, and yellowness b* in five replications each [13,30].

### 2.10. Statistical Analysis

Statistica 13.3 software (StatSoft Inc., Tulsa, OK, USA) was used to analyze the results. The obtained data were analyzed with one-way (ANOVA) analysis of variance followed by the post hoc test with the Fisher’s LSD (least significant difference) at the significance level of α = 0.05 to assimilate mean values.

## 3. Results and Discussion

### 3.1. Pasting Characteristics of Raw Materials Blends

Pasting characteristics may be useful for the indirect estimation of the extrusion-cooking treatment intensity of raw materials with significant content of starch. Peak viscosity indicates the ability of raw materials composition to water absorption and starch swelling and is influenced mostly by starch content in blends. Based on the results of the pasting profile of tested compositions (Table 1), increasing the amount of cricket flour reduced peak viscosity due to the lower amount of starch coming from wheat and corn flour. It suggests that replacing the starchy components with high-protein cricket flour gives lower water absorption and swelling capacity, which may have an effect on the insufficient treatment of blends with low starch content (see Table 2). Much lower PVs were observed in extruded products, such as fresh carrot pulp-supplemented pellets and snacks [40] or extruded beans [32].

Hot paste viscosity (HPV) indicates the sensitivity of the swollen starch granules to shearing and disintegration. Mitrus et al. [32] reported 107–130 mPa s in unprocessed beans of various origins and much lower values in extruded beans. Wheat–corn control blend showed twice as higher HPV than the blends with substitution of starchy components with cricket flour at 30% level. Increasing the amount of cricket flour in blends caused a lowering of the cold paste viscosity values and thus, gel formation ability due to the higher protein and fiber contents replacing starch and possible interaction between the starch and other components occurring after the heating stage. It may suggest that a higher temperature is required to transform extruded blends with cricket flour addition, which was confirmed by the gelatinization temperature measured during raw materials heating. The temperature profile during the extrusion-cooking of snack pellets was enough to gelatinize the starch and transform the materials into the viscous stage. Breakdown (BD) demonstrates the stability of the paste during cooking, so the increased level of cricket flour significantly lowered the susceptibility of starch granules to disintegrate during treatment. Setback (SB) results that were twice as low were recorded in samples with 30% of cricked flour, showing a lower retrogradation tendency that was connected with a lower amount of starch in the total sample mass as confirmed by proximate composition, so further syneresis in these samples may be lower. 

### 3.2. The Basic Composition and Fatty Acids Profile of Snack Pellets

First, the proximate composition and fatty acids profile of the selected extrudates were determined (Table 2 and Table 3). Both the basic composition and the fatty acid profile of the produced snacks differed significantly. The dissimilarities were mainly due to the different content of the cricket flour applied as an additive in the processing of the snack pellets extrudates. 

Application of 30% of cricket flour to the extruded recipes increased the content of basic chemical components, such as crude protein, crude ash, crude fat, and crude fiber, in comparison to control samples and products containing 10% of insect flour, for both screw speeds (60 and 100 rpm) applied during processing. 

The high content of total protein–a valuable dietary ingredient for humans, in insects, has been described in many studies [4,41,42,43]. The results indicate high protein content (45–70%), fiber (5%), and lipids (20–30%) in cricket flour [43]. Confirmation of the high value of cricket flour can be results presented by Stone et al. [12], which revealed 65.5% and 66% of protein content in cricket and mealworm, respectively. Important is high digestibility and oil and water absorption capacity, which is observed in the case of protein extracts obtained from edible insects. Moreover, cricket proteins were proven that they have the ability to form gels [44]. This property may be of importance for technological and functional applications of edible insect flours in food processing [12]. The observed increase in the total protein content of snacks with the high addition of insect flour clearly resulted in an increase in their nutritional value. In our research, the average total protein content of snacks containing 30% insect flour was 54.54 and 162.74% higher in comparison to snack pellets with 10% of this component and to the control sample. In control snack pellets without an insect flour addition, the protein content was the lowest.

In the control sample, the protein content reached 9.18–9.33 g/100 g, ash content was 2.85–2.91 g/100 g, ether extract was 0.33–0.80 g/100 g, fiber was 0.00 g/100 g, and carbohydrate content was 76.99–77.39 g/100 g. The highest levels of proximate components were found in snack pellets supplemented with 30% of insect flour additions. Here, protein content reached 24.07–24.60 g/100 g, fat 0.12–0.36 g/100 g, ash 4.19–5.64 g/100 g, crude fiber 5.92–7.22 g/100 g, and carbohydrates at 71.02–75.55 g/100 g (Table 2). Ramírez-Rivera et al. [45] found that application of the increased level of grasshopper meal up to 40 g/100 g lowered carbohydrates content (89.45–65.39 g/100 g) but simultaneously increased protein (5.33–22.51 g/100 g) and ash (1.67–7.88 g/100 g) contents. In addition, Ribeiro et al. [30] observed increased content of proteins in insect-rich snacks when house cricket (*Acheta domesticus*) was included in extruded snacks, while García-Segovia et al. [46] found insect flour to be a good source of protein and minerals in extruded snacks.

Our research showed that increasing the content of cricket flour as a component in the production of extruded snack pellets from 10 to 30% did not significantly affect the PUFA content while increasing the SFA content in the total fatty acids profile (Table 3). 

This is comparable to the outcome of the work of Raksakantong et al. [47], who noted edible insects as a good provenance fatty acids as long-chain omega-3 fatty acids characterized with healthy and beneficial features, such as eicosapentaenoic acid, alpha-linolenic acid, and other polyunsaturated fatty acids, and thus making allowance for them as a good source of proteins and valuable lipids. The absence of some fatty acids in extruded products could be the effect of the formation of complexes of protein-lipids and starch lipids which may be less extractable during measurements.

The lower-than-expected crude fiber content in extruded snack pellets could be caused by the process of degradation of macromolecular compounds as a result of the extrusion-cooking process and the related impact of high temperature and pressure on the raw material [48,49]. Still, the extrusion process can reduce the content of bioactive substances while often increasing their bioavailability in the human body, which translates into extending the actual volume of absorbed bioactive compounds [50].

Based on the performed study, insect flour can be inferred as a component with great potential to influence the nutritional value of snack foods due to its high protein and fiber content in snack pellets.

### 3.3. Free Radical Scavenging Activity and Phenolic Compounds Content of Snack Pellets Enriched with Cricket Flour

Total phenolics content (TPC), as assessed via the Folin–Ciocalteu method, is a helpful parameter for determining the antioxidant potential of plants or foodstuffs extracts. The assay is used to estimate the amount of contained high active antioxidants (polyphenols The addition of cricket flour significantly increased TPC in the analyzed samples compared to wheat–corn base control samples. It was also confirmed that the TPC increased with increasing cricket flour content in snack pellets (Table 4). Production parameters have also an influence on total phenolic content (Table 4). Study results indicate that 36% of moisture content and 100 rpm screw rotation speed as better for the production of snack pellets with 10% of cricket flour content. In the case of 30% of cricket flour content in snack pellets, the most valuable turned out to be 32% of moisture content and 60 rpm screw rotation speed. 

Spectrophotometric DPPH assay allowed us to determine the ability of the analyzed extracts of snack pellets to scavenge free radicals. The results explicitly indicate the high ability of the extracts to scavenge free radicals. This was above 92% for both amounts of cricket flour additive (Table 4). The scavenging of DPPH free radicals was extremely rapid, and we saw activity at the level above 40% and 50% just after the first minute of the experiment. The obtained results are presented as Trolox equivalent antioxidant activity (TEAC). The value was calculated for all analyzed samples at 15 min from reaction initiation.

**Figure 2:**
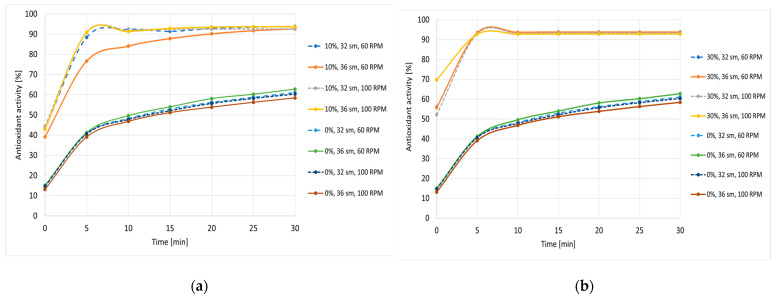
Antioxidant activity obtained for snack pellets prepared under various processing parameters: initial moisture (sm) and screw rotation speed (RPM): (**a**) control and 10% of cricket flour; (**b**) control and 30% of cricket flour.

An important part of the research was the analysis of the influence of production parameters on the activity of the tested samples. Our results indicated that extrusion conditions significantly impacted the nutritional quality of the functional food products. Figure 2 presents a comparison of the antioxidant activity of snack pellets prepared at various extrusion parameters. As can be noted, there were no significant differences in antioxidant activity depending on the production conditions after the first 10 min of the reaction. A more evident impact, however, was observed if 10% of cricket flour addition was used but only if 36% of initial moisture and 60 rpm screw speed was applied during processing-for these samples, the antioxidant activity was slightly weaker.

In the case of 30% of cricket flour, the production parameters significantly impacted the activity during the first 10 min from reaction initiation. In the next step of the reaction, the production parameters did not have such an important influence on the free radical scavenging activity of the samples. No significant differences were found in antioxidant activity for the various parameters, whereas in the case of TPC, this influence was more significant. Nevertheless, in both cases, the production parameters did not have a destructive impact on the analyzed samples. Edible insects may provide biologically active compounds to consumers, including antioxidants. Around the mid-20th century, scientists discovered the presence of phenolic compounds in insect cuticles, wings, and intestinal tracts [51]. During sclerotization (the process of hardening the epidermis of insects), the incorporation of phenolic compounds in the cuticular matrix occurs with the involvement of structural proteins and chitin [52]. The phenolics found in edible insects varied among insect species because of different types of feed consumed and different biosynthesis per specie [53].

Considering the obtained study results, there is evidence of high activity of snack pellet half-products enriched with cricket flour. In our study, both amounts of cricket flour addition significantly improved free radical scavenging activity, along with the increased total phenolics content. Such an outcome is significant due to the fact that the secondary polyphenols have not only antioxidant activity but also various other pro-health properties, including anti-inflammatory and antimicrobial activities [17]. 

In our experiment, the addition of cricket flour increased the content of polyphenols in the newly developed snack pellets, similar to the study concerning gluten-free bread enriched with cricket that was conducted by Kowalczewski et al. [54]. The analyzed antioxidant activity was also enhanced due to the addition of cricket flour. 

In the course of our research, the antioxidant potential of cricket flour-enriched snack pellets extracts were evaluated via DPPH radical assay. The outcome confirmed the promising antioxidant capacity of the product. Our results can be compared with the results presented by Suh et al. [55], based on the study of the antioxidant activity of extracts obtained from the Japanese rhinoceros beetle (*A. dichotoma*). These authors reported moderate scavenging action depending on the extraction solvent, with the lowest IC_50_ (0.119 mg/mL) for the methanolic extract. In contrast, aqueous extracts of *H. parallela* revealed significant antioxidant activity (IC5_0_ of 1.45 mg/mL) [56]. In other work, data have also appeared indicating a correlation between TPC and DPPH for *T. molitor* and *A. domesticus* extracts, which showed a clear relationship between a higher content of polyphenols and a higher scavenging capacity of DPPH [57]. In the case of our research, the polyphenols content and the antioxidant activity of the snack pellets were found to be also positively correlated with the addition of cricket flour (Table 5). Similar observations were found by Zielińska et al. [13] when testing antioxidant properties of insect flours and muffins with mealworm (*Tenebrio molitor*) and cricket (*Gryllodes sigillatus*) flour based on the determination of total phenolic content (TPC) and the ability to neutralize DPPH· and ABTS· free radicals. They also found that this fortification resulted in a lower predicted glycemic index, which makes the addition of insect flour favorable in products for people who often struggle with various conditions, such as obesity, diabetes, and insulin resistance [13]. 

Previously, the antioxidant activity of insects was associated only with the presence of polyphenols. However, bioactive peptides, proteins, and free amino acids coming from insects may also redound to the antioxidant activity of the tested extracts [17,55,58]. Folin–Ciocalteu reagent is not selective to phenolics and uses tungsten ions to oxidize other compounds, such as amino acid residues. It can be concluded that this activity probably results from the cooperation of the whole group of bioactive molecules, not only polyphenols. In addition, antioxidant activity may be due to synergistic interaction between polyphenols and proteins [17,55].

The increase in the antioxidant capacity of snack pellets may come from the presence of the active compounds in them and from the method of cricket farming and cricket product preparation. According to Zielińska et al. [59], the thermal treatment of insects may significantly enhance their biological activity. Furthermore, an additional increase in activity may also be the result of the process of enzymatic hydrolysis, which is analogous to digestion in the human digestive tract [60]. As well, the influence of the intestinal microflora may enlarge the antioxidant potential of the digested products [61]. Taking into account the complexity of the metabolic processes taking place in the large intestine, this section on the nutritional properties of the cricket product cannot be omitted. 

Therefore, an important part of further research will be to confirm the absorption of these secondary plant metabolites from food, and the effect of these compounds on the composition of products enriched with insects. Studies on the bioavailability of compounds can significantly increase interest in entomophagy. The new knowledge will be an added value in favor of consuming insects or insect-enriched foods. 

### 3.4. WAI and WSI of Snack Pellets Enriched with Cricket Flour

The results of the Water Absorption Index (WAI) and Water Solubility Index (WSI) of snack pellets enriched with cricket flour are presented in Table 6. The WAI indicates the intensity of the thermomechanical treatment during the extrusion cooking process and reveals the amount of gelatinized starch able to absorb and hold the water after centrifugation [38]. For the control sample based on wheat-corn recipes, the water absorption ranged from 3.15 g/g at the lowest initial moisture content and when minimal screw speed was used to 4.43 g/g for samples extruded at the highest rpm and at 36% of initial moisture content (Table 6).

In snack pellets samples, with the addition of cricket flour, a lowering of WAI was noted. This is probably related to the increased protein content and, thus, decreased the total carbohydrates content (from 76.99–77.39% in the control sample to 52.16–52.40% when the insect flour content was 30% in the recipe. A lowered content of carbohydrates, especially starch, is responsible for lowered WAI, and this has a connection to low starch gelatinization. Application of cricket flour at the level of 10 and 30% showed a significant decrease in WAI, especially when low moisture was used, and low screw speed was applied during snack pellets extrusion. We observed that while screw speed had an effect, strong dependencies were also observed when varied initial moisture levels were employed. Here, increased water level availability for transforming and gelatinizing the starch in all tested samples increased water absorption independently of screw speed and insect flour content. This means that the water added to raw materials had a crucial role in starch gelatinization and, thus, in WAI in the tested samples. A low WAI level in the snack pellets indicates the degree of starch gelatinization and can affect the expansion procedure, especially without frying. This effect may be notable, especially in the microwave or hot air processing [62]. The WAI in snack pellets enriched with cricket flour was lower than in snack pellets or microwave-expanded snacks based on potato components with fresh carrot pulp addition [40]. In that work, the WAI of snack pellets varied from 3.71 to 4.85 g/g, while expanded snacks showed WAI ranged from 4.45 to 5.61 g/g with ambiguous effects of processing conditions. Herein, lower levels of WAI in such potato-based products with carrot addition may be connected with higher amounts of starch in composition available to gelatinize at lower temperatures than wheat–corn-based blends with high protein cricket flour additive. 

Lowering the starch content in the recipe by the application of cricket flour also affected the WSI results. WSI allows us to measure soluble components not compacted within the starch-protein matrix formed during the extrusion-cooking and results mostly from starch degradation but also from the presence of non-soluble components in the recipe. The results of WSI obtained for the snack pellets of our experiment showed similar tendencies when no and 10% of cricket flour was added into the wheat–corn-based recipe (Table 6). Higher WSI was noted with an increased level of initial moisture content and revealed the significant effect of the screw speed applied during snack pellets processing. The same trend was found when 30% of insect flour was used, and, in this case, the results were much greater. Accordingly, WSI increased with increasing moisture and with higher screw speeds in snack pellets with the highest content of cricket flour, hence indicating that components were not sufficiently integrated into the pellet structure-probably due to the higher content of crude fiber and the lowered amount of starch needed to combine all the components (note: because they are unprocessable at low temperature, fiber fractions can be easy rinsed out from the snack pellet sample). The results of WSI for these samples were greater than 30%, which suggests insufficient combining and processing of ingredients under the conditions applied in the experiment. Regarding comparative work, Téllez-Morales et al. [63] tested the addition of 8–40% of cricket flour into extruded nixtamalized corn flour compositions and found optimum cricket flour concentration at 31.9% at a die temperature of 145.6 °C and moisture level of 19.7% when used in the processing of corn-based snacks with acceptable characteristics. Moreover, García-Segovia et al. [46] found that ash content was significantly related both to WAI and WSI of snacks with 5% of two types of insect flours added. Lisiecka et al. [40] found much lower WSI results of potato-based snack pellets supplemented with fresh carrot pulp, probably due to the lower starch gelatinization temperature of potato-based components and lower amount of protein, ash, or fiber in applied vegetable additive. 

### 3.5. Texture of Snack Pellets

The texture of snack pellets evaluated as the cutting force is presented in Table 6 if different processing variables or additive content was used. Samples of pellets prepared with a control recipe based on wheat and corn components showed the highest hardness ranging from 25.01 to 33.01 N in samples processed at 60 rpm; more uniform results were noted when 100 rpm was applied during processing. The addition of 10% of cricket flour significantly lowered the hardness of snack pellets, especially when a lower initial moisture level was applied and the cutting force was 13.32–15.78 N. Much lower results were found in pellets supplemented with the highest amount of cricket flour; three times less force were needed to cut the snack pellet sample compared to the control ones. It suggests that increasing the amount of edible insect flour is responsible for loosening the internal structure of extruded pellets due to the increased amount of non-starchy components affecting disruption of the internal molten starch matrix. This loosened structure confirmed also by the low results of pasting characteristics, especially PV, and the high-water solubility results of these samples, made snack pellets more fragile and less hard. It may have consequences in pellets stability during storage because if samples have more delicate internal structure and lower hardness, they are more susceptible for damage during transportation of storage. On the other hand, this loose structure may be beneficial information of the delicate structure after frying, microwaving, or hot air expanding due to the less compacted structure of pellets, but it was tested in a separate study. Lisiecka et al. [64] tested the cutting force of potato-based pellets and fried snacks with the addition of leek and onion, and they found the cutting force values higher (77.63–102.69 N for control and 20.91–146.79 N for supplemented pellets) than obtained for wheat–corn pellets supplemented with cricket flour. Similarly, results presented by Lisiecka and Wójtowicz [65] for snack pellets based on a potato recipe with the addition of up to 30% of fresh beetroot pulp showed higher breaking forces tested with a 3-point bending test than observed in wheat-corn supplemented with edible cricket flour assessed in the cutting test.

### 3.6. Results of Snack Pellets Color

The results of color coordinate and the total color change are presented in Table 7. All samples with addition from 10% to 30% were darker than control pellets, and lower L* values were observed with any significant differences in lightness due to processing conditions. The changes in the snack pellets’ color mainly resulted from the increase in the content of cricket flour characterizing brown color coming from insect carapace [13]. The lowest redness a* was noted in control samples processed at 60 rpm. Slight changes were observed in the red tint of control snack pellets processed at 100 rpm and when 10 and 30% of cricket flour were applied, not dependently on the processing conditions. Snack pellets containing 30% of cricket flour were less intensively yellow than those supplemented with 10% of edible insects, and double lower yellowness intensity was noted as compared to control samples. b* values were found from 24.38 for the control sample, which was yellow due to the presence of corn flour up to 12.45 at the highest level of cricket addition replacing yellow components and becoming browner then yellow. In this case, slightly more intensive yellow tint was observed in samples processed at higher screw speeds and higher initial moisture content. All these color indicators had great influence on the total color change index *ΔE*, when it is above 3, it means that color can be easily differentiated by eyes from the reference sample [40].

Application of both levels of cricket flour significantly changed the total color of snack pellets, with values of *ΔE* ranging from 13.10 to 21.88. Much lower changes in color were found by Lisiecka et al. [40] testing snack pellets and crisps with fresh carrot pulp addition due to the light color of all components; *ΔE* was max 2.16 in pellets and 7.97 after microwave expansion. However, the total color change may be an effect of other processes occurring during the extrusion-cooking, such as Maillard reactions, caramelization, or degradation of pigments [66].

## 4. Conclusions 

The cricket flour addition in wheat-corn blends had a significant impact on the proximate composition of snack pellets processed with the extrusion-cooking. Enhanced levels of protein from 9.3 to 24.6 g/100 g were found if cricket flour content was boosted from 10 to 30% of the total components in the recipe. Indeed, an almost triple increase in crude fiber was noted if insect flour was used at a 30% level. Processing conditions showed a slight effect on the chemical composition of extruded snack pellets. Cricket flour content at either 10 or 30% of total flour content had a significant effect on snack pellets WAI and WSI. Here, the WAI decreased at low initial moisture and low screw speed during snack pellets extrusion. Cricket flour addition lowered snack pellets’ WAI and lightness, while WSI increased with increasing initial moisture and with higher screw speeds and the highest cricket flour addition. This indicates that components were not sufficiently integrated into the snack pellets structure. A similar conclusion can be stated based on cutting force results, which were significantly lower with an increased amount of cricket flour in the recipe. Such an outcome was probably due to high crude protein and crude fiber content and, thus, low starch content. Moreover, results showed that cricket flour addition significantly increased total polyphenols content in comparison to the wheat–corn control sample. The antioxidant activity of snack pellet extracts increased with increasing cricket flour content in the recipe. Therefore, it can be concluded that cricket flour shows great potential to enhance the nutritional value of newly developed food products due to the high protein and fiber content in snack pellets.

## Figures and Tables

**Table 1:** Pasting characteristics of raw materials blends before extrusion depends on the cricket flour level.

Recipe	Gelatinization Temperature [°C]	PV [mPas]	HPV [mPas]	CPV [mPas]	BD [mPas]	SB [mPas]
Control blend	73.0 ± 0.9 ^a^	472 ± 2 ^c^	354 ± 4 ^c^	666 ± 2 ^c^	118 ± 2 ^c^	312 ± 2 ^c^
10% cricket flour	74.7 ± 1.2 ^a^	369 ± 3 ^b^	284 ± 5 ^b^	582 ± 3 ^b^	85 ± 2 ^b^	298 ± 2 ^b^
30% cricket flour	80.7 ± 0.7 ^b^	197 ± 1 ^a^	156 ± 2 ^a^	303 ± 4 ^a^	41 ± 3 ^a^	147 ± 4 ^a^

PV—peak viscosity, HPV—hot paste viscosity, CPV—cold paste viscosity, BD—breakdown, SB—setback; ^a–c^—means indicated with similar letters in columns do not differ significantly at α = 0.05.

**Table 2:** Basic composition of snack pellets depends on the additive level and processing variables (n = 3, ±SD).

Cricket Flour Content [%]	Screw Speed [rpm]	Moisture Content [%]	Component [g/100 g]				
			Crude Protein	Crude Ash	Crude Fat	Crude Fiber	Carbohydrates
0	60	32	9.33 ± 0.87 ^a^	2.90 ± 0.84 ^a^	0.42 ± 0.06 ^a^	0.00 ± 0.00 ^a^	77.20
		36	9.18 ± 0.90 ^a^	2.88 ± 0.72 ^a^	0.33 ± 0.04 ^a^	0.00 ± 0.00 ^a^	77.39
	100	32	9.21 ± 0.88 ^a^	2.91 ± 0.68 ^a^	0.41 ± 0.04 ^a^	0.00 ± 0.00 ^a^	77.38
		36	9.31 ± 0.92 ^a^	2.85 ± 0.76 ^a^	0.80 ± 0.06 ^b^	0.00 ± 0.00 ^a^	76.99
10	60	32	13.53 ± 1.12 ^b^	3.04 ± 0.78 ^a^	2.54 ± 0.42 ^b^	0.64 ± 0.06 ^b^	70.47
		36	14.56 ± 1.16 ^b^	2.99 ± 0.76 ^a^	1.95 ± 0.46 ^b^	0.95 ± 0.08 ^c^	69.92
	100	32	14.44 ± 1.18 ^b^	3.04 ± 0.72 ^a^	3.33 ± 0.58 ^b^	0.98 ± 0.12 ^c^	68.53
		36	14.72 ± 1.08 ^b^	3.05 ± 0.82 ^a^	1.26 ± 0.28 ^b^	0.93 ± 0.14 ^c^	70.24
30	60	32	24.07 ± 1.66 ^c^	3.29 ± 0.84 ^a^	8.28 ± 0.96 ^c^	3.24 ± 0.36 ^d^	52.16
		36	24.54 ± 1.62 ^c^	3.10 ± 0.76 ^a^	6.01 ± 0.68 ^c^	3.30 ± 0.42 ^d^	54.18
	100	32	24.12 ± 1.54 ^c^	3.23 ± 0.84 ^a^	7.77 ± 0.82 ^c^	3.02 ± 0.38 ^d^	53.09
		36	24.60 ± 1.62 ^c^	3.22 ± 0.92 ^a^	7.76 ± 0.78 ^c^	3.27 ± 0.44 ^d^	52.40

^a–d^—means indicated with similar letters in columns do not differ significantly at α = 0.05.

**Table 3:** Fatty acid profiles of selected snack pellets depend on the additive level and processing variables (n = 3, ±SD).

Fatty Acids	Extrudates							
	10% Cricket Flour				30% Cricket Flour			
	32% m.c.		36% m.c.		32% m.c.		36% m.c.	
	60 rpm	100 rpm	60 rpm	100 rpm	60 rpm	100 rpm	60 rpm	100 rpm
Butyric acid C 4:0	nd	0.049 ± 0.008 ^a^	0.052 ± 0.008 ^a^	nd	nd	nd	nd	0.045 ± 0.006 ^a^
Caproic acid C 6:0	nd	nd	nd	nd	nd	nd	nd	0.026 ± 0.004 ^a^
Caprylic acid C 8:0	nd	nd	nd	nd	nd	nd	0.044 ± 0.008 ^a^	0.057 ± 0.008 ^a^
Lauric acid C 12:0	nd	nd	nd	0.063 ± 0.010 ^a^	nd	0.053 ± 0.008 ^a^	0.058 ± 0.006 ^a^	0.059 ± 0.006 ^a^
Myrystic acid C 14:0	0.328 ± 0.022 ^a^	0.370 ± 0.030 ^a^	0.330 ± 0.028 ^a^	0.394 ± 0.034 ^a^	0.472 ± 0.036 ^b^	0.494 ± 0.038 ^b^	0.480 ± 0.034 ^b^	0.471 ± 0.036 ^b^
Pentadecylic acid C 15:0	0.069 ± 0.006 ^a^	0.066 ± 0.004 ^a^	0.086 ± 0.006 ^b^	0.112 ± 0.008 ^c^	0.085 ± 0.008 ^b^	0.073 ± 0.006 ^a^	0.113 ± 0.008 ^c^	0.065 ± 0.004 ^a^
Palmitic acid C 16:0	19.135 ± 1.624 ^a^	19.596 ± 1.686 ^a^	18.964 ± 1.652 ^a^	20.051 ± 1.722 ^a^	23.888 ± 1.746 ^b^	24.630 ± 1.786 ^b^	24.197 ± 1.742 ^b^	24.669 ± 1.766 ^b^
Palmitoleic acid C 16:1 n-9	0.401 ± 0.032 ^a^	0.429 ± 0.038 ^a^	0.426 ± 0.042 ^a^	0.412 ± 0.040 ^a^	0.529 ± 0.042 ^a^	0.604 ± 0.048 ^a^	0.539 ± 0.038 ^a^	0.547 ± 0.042 ^a^
Palmitoleic acid C 16:1 n-7	nd	nd	nd	0.089 ± 0.008 ^a^	nd	nd	nd	nd
Heptadecanoic acid C 17:0	0.170 ± 0.016 ^a^	0.157 ± 0.014 ^a^	0.154 ± 0.008 ^a^	0.143 ± 0.012 ^a^	0.167 ± 0.018 ^a^	0.198 ± 0.022 ^a^	0.182 ± 0.016 ^a^	0.188 ± 0.014 ^a^
Stearic acid C 18:0	5.984 ± 0.522 ^a^	6.261 ± 0.584 ^a^	6.076 ± 0.592 ^a^	6.441 ± 0.576 ^a^	8.238 ± 0.722 ^b^	8.359 ± 0.728 ^b^	8.216 ± 0.742 ^b^	8.571 ± 0.718 ^b^
Oleic acid C 18:1 n-9	36.174 ± 3.080 ^b^	33.852 ± 3.120 ^b^	35.657 ± 2.982 ^b^	32.356 ± 3.128 ^b^	31.330 ± 3.068 ^b^	30.325 ± 3.144 ^b^	32.368 ± 2.824 ^b^	32.773 ± 3.146 ^b^
Linoleic acid C 18:2 n-6	34.405 ± 3.222 ^a^	35.954 ± 3.256 ^a^	34.891 ± 3.842 ^a^	37.182 ± 3.628 ^a^	33.137 ± 3.422 ^a^	32.740 ± 2.980 ^a^	31.668 ± 2.864 ^a^	30.607 ± 3.022 ^a^
α-Linolenic acid C 18:3 n-3	2.286 ± 0.249 ^b^	2.314 ± 0.264 ^b^	2.277 ± 0.212 ^b^	2.024 ± 0.196 ^b^	1.582 ± 0.125 ^a^	1.986 ± 0.167 ^b^	1.503± 0.159 ^a^	1.367 ± 0.122 ^a^
Arachidic acid C 20:0	0.440 ± 0.044 ^a^	0.400 ± 0.036 ^a^	0.420 ± 0.038 ^a^	0.380 ± 0.032 ^a^	0.345 ± 0.040	0.334 ± 0.036 ^a^	0.374 ± 0.033 ^a^	0.356 ± 0.035 ^a^
Gondoic acid C 20:1 n-9	0.424 ± 0.042 ^b^	0.401 ± 0.038 ^b^	0.422± 0.044 ^b^	0.353 ± 0.036 ^b^	0.227 ± 0.028 ^a^	nd	0.258 ± 0.018 ^a^	0.200 ± 0.022 ^a^
Eicosadienoic acid C 20:2 n-6	nd	nd	nd	nd	nd	0.079 ± 0.008 ^a^	nd	nd
Behenic acid C 22:0	0.185 ± 0.012 ^c^	0.152 ± 0.014 ^b^	0.245 ± 0.018 ^d^	nd	nd	0.125 ± 0.008 ^a^	nd	nd
Total [%]	100.00	100.00	100.00	100.00	100.00	100.00	100.00	100.00
ΣSFA [% in total]	26.311	27.051	26.327	27.584	33.195	34.265	33.664	34.507
ΣMUFA [% in total]	36.999	34.682	36.505	33.121	32.085	30.929	33.165	33.520
ΣPUFA [% in total]	36.690	38.267	37.168	39.205	34.719	34.805	33.171	31.973

^a–d^—means indicated with similar letters in rows do not differ significantly at α = 0.05; ΣSFA—sum of saturated fatty acids; ΣMUFA—sum of monounsaturated fatty acids; ΣPUFA—sum of polyunsaturated fatty acids; nd—not detected.

**Table 4:** Total phenolics content, DPPH scavenging activity, and TEAC value of snack pellets depend on additive level and processing variables (n = 3; ±SD).

Cricket Flour Content [%]	Screw Speed [rpm]	Moisture Content [%]	TPC [μg GAE/g Dry Weight]	DPPH Scavenging Activity [%]	TEAC Value [µg/g Product]
0	60	32	62.1 ± 1.2 ^a^	50.11 ± 0.18 ^a^	111.45 ± 4.38 ^a^
		36	60.6 ± 1.9 ^a^	51.02 ± 0.23 ^b^	113.08 ± 5.12 ^a^
	100	32	78.1 ± 2.1 ^b^	50.28 ± 0.22 ^a^	106.54 ± 6.11 ^a^
		36	60.8 ± 1.8 ^a^	51.01 ± 0.35 ^b^	103.50 ± 4.98 ^a^
10	60	32	86.3 ± 3.2 ^c^	93.83 ± 0.11 ^d^	160.63 ± 7.56 ^b^
		36	93.0 ± 4.3 ^d^	92.65 ± 0.23 ^c^	156.43 ± 6.98 ^b^
	100	32	84.9 ± 1.2 ^c^	92.57 ± 0.34 ^c^	162.97 ± 8.02 ^b^
		36	119.8 ± 6.4 ^e^	93.67 ± 0.17 ^d^	163.43 ± 7.32 ^b^
30	60	32	152.0 ± 4.4 ^g^	93.75 ± 0.28 ^d^	164.14 ± 9.02 ^b^
		36	137.2 ± 2.8 ^f^	93.85 ± 0.21 ^d^	164.25 ± 7.78 ^b^
	100	32	146.5 ± 5.4 ^g^	93.38 ± 0.62 ^d^	163.79 ± 8.99 ^b^
		36	114.5 ± 3.3 ^e^	92.85 ± 0.29 ^c^	163.08 ± 8.09 ^b^

^a–g^—means indicated with similar letters in columns do not differ significantly at α = 0.05; TPC—total phenolics content; DPPH—scavenging of DPPH free radicals; TEAC—Trolox equivalent antioxidant activity.

**Table 5:** Pearson’s correlation coefficients depend on additive content at α = 0.05.

Sample Parameters	TPC	DPPH	TEAC
60 rpm, 32% mc	0.997	0.788	0.794
60 rpm, 36% mc	0.995	0.790	0.764
100 rpm, 32% mc	0.907	0.874	0.841
100 rpm, 36% mc	0.779	0.756	0.752

TPC—total phenolics content; DPPH—scavenging of DPPH free radicals; TEAC—Trolox equivalent antioxidant activity.

**Table 6:** Results of WAI, WSI, and cutting force of snack pellets depend on additive level and processing variables enriched with insect flour (^1^ n = 3, ^2^ n = 5 ± SD).

Cricket Flour Content [%]	Screw Speed [rpm]	Moisture Content [%]	WAI ^1^ [g/g]	WSI ^1^ [%]	Cutting Force ^2^ [N]
0	60	32	3.15 ± 0.32 ^b^	10.34 ± 0.52 ^b^	33.01 ± 2.89 ^d^
		36	3.82 ± 0.28 ^b^	12.47 ± 0.55 ^c^	25.01 ± 1.52 ^c^
	100	32	3.43 ± 0.22 ^b^	14.14 ± 0.49 ^d^	30.79 ± 2.94 ^d^
		36	4.43 ± 0.18 ^c^	14.72 ± 0.46 ^d^	31.32 ± 2.96 ^d^
10	60	32	2.46 ± 0.12 ^a^	8.09 ± 0.28 ^a^	15.78 ± 2.21 ^b^
		36	2.35 ± 0.14 ^a^	8.81 ± 0.32 ^b^	25.27 ± 1.72 ^c^
	100	32	3.75 ± 0.28 ^b^	8.70 ± 0.36 ^b^	13.32 ± 4.21 ^ab^
		36	3.70 ± 0.30 ^b^	9.27 ± 0.46 ^b^	22.63 ± 3.54 ^c^
30	60	32	2.65 ± 0.14 ^a^	18.24 ± 0.62 ^e^	11.82 ± 2.13 ^a^
		36	2.96 ± 0.12 ^b^	28.73 ± 0.86 ^f^	10.98 ± 1.15 ^a^
	100	32	3.55 ± 0.18 ^b^	27.27 ± 0.76 ^f^	11.96 ± 1.63 ^a^
		36	3.52 ± 0.22 ^b^	32.04 ± 0.96 ^g^	14.73 ± 3.30 ^b^

^a–g^—means indicated with similar letters in columns do not differ significantly at α = 0.05; WAI—Water Absorption Index; WSI—Water Solubility Index.

**Table 7:** Color profile of extruded snack pellets supplemented with cricket flour processed under various conditions (n = 5 ± SD).

Cricket Flour Content [%]	Screw Speed [rpm]	Moisture Content [%]	Color Parameters			
			L*	a*	b*	*ΔE*
0	60	32	84.46 ± 0.98 ^d^	3.86 ± 0.18 ^a^	21.51 ± 0.59 ^d^	ref
		36	83.65 ± 0.56 ^d^	4.09 ± 0.23 ^a^	22.10 ± 1.10 ^d^	ref
	100	32	81.56 ± 0.43 ^d^	5.07 ± 0.03 ^cd^	24.18 ± 0.45 ^e^	ref
		36	81.83 ± 0.40 ^d^	5.09 ± 0.11 ^cd^	24.38 ± 0.37 ^e^	ref
10	60	32	68.35 ± 1.47 ^c^	4.75 ± 0.35 ^bc^	13.88 ± 0.89 ^bc^	17.87 ± 1.49 ^b^
		36	67.88 ± 0.96 ^bc^	4.55 ± 0.20 ^b^	13.85 ± 0.44 ^bc^	17.49 ± 1.00 ^b^
	100	32	68.47 ± 2.72 ^c^	4.84 ± 0.27 ^bc^	14.60 ± 0.32 ^bc^	17.47 ± 2.52 ^b^
		36	72.88 ± 1.65 ^e^	4.73 ± 0.15 ^bc^	14.88 ± 0.33 ^c^	13.10 ± 1.22 ^a^
30	60	32	64.59 ± 2.23 ^a^	5.11 ± 0.20 ^cd^	12.45 ± 0.75 ^a^	21.88 ± 2.28 ^c^
		36	65.17 ± 1.01 ^ab^	5.45 ± 0.12 ^d^	13.47 ± 0.44 ^ab^	19.92 ± 1.06 ^bc^
	100	32	65.97 ± 1.30 ^abc^	5.44 ± 0.15 ^d^	13.48 ± 0.44 ^ab^	20.22 ± 1.33 ^bc^
		36	65.85 ± 1.60 ^abc^	5.49 ± 0.23 ^d^	13.49 ± 0.66 ^ab^	19.36 ± 1.66 ^bc^

L*—lightness from 0(black) to 100 (white); a*—balance between red(+) and green(−); b*—balance between yellow(+) and blue(−); *ΔE*—total color change index; ^a–e^—means indicated with similar letters in columns do not differ significantly at α = 0.05.

## Data Availability

All of the data is contained within the article.
